# Supplementary material for: Prussian blue analog with separated active sites to catalyze water driven enhanced catalytic treatments
Source: Nat Commun. 2023 Aug 5;14:4709. doi: 10.1038/s41467-023-40470-z (PMC10404294; doi:10.1038/s41467-023-40470-z)
Supplement: Supplementary file 1 — Supplementary_Information [file 41467_2023_40470_MOESM1_ESM.pdf]

Supplementary Information

Prussian blue analog with separated active sites to catalyze water driven enhanced catalytic treatments

Liu-Chun Wang[1], Pei-Yu Chiou[1], Ya-Ping Hsu[1], Chin-Lai Lee[2], Chih-Hsuan Hung[1], Yi-Hsuan Wu[1], Wen-Jyun Wang[3], Gia-Ling Hsieh[1], Ying-Chi Chen[1], Li-Chan Chang[4], Wen-Pin Su[4,5], Divinah Manoharan[1], Min-Chiao Liao[2], Suresh Thangudu[6], Wei-Peng Li[3,7,8,*], Chia-Hao Su[2,6,9,10,11,*], Hong-Kang Tian[12,*], Chen-Sheng Yeh[1,8,*]

[1]Department of Chemistry, National Cheng Kung University, Tainan 701, Taiwan
[2]Department of Diagnostic Radiology, Kaohsiung Chang Gung Memorial Hospital, Kaohsiung 833, Taiwan
[3]Department of Medicinal and Applied Chemistry, Kaohsiung Medical University, Kaohsiung 807, Taiwan
[4]Institute of Clinical Medicine, College of Medicine, National Cheng Kung University, Tainan 704, Taiwan
[5]Departments of Oncology and Internal Medicine, National Cheng Kung University Hospital, College of Medicine, National Cheng Kung University, Tainan 704, Taiwan
[6]Center for General Education, Chang Gung University, Taoyuan, 333, Taiwan
[7]Drug Development and Value Creation Research Center, Kaohsiung Medical University, Kaohsiung 807, Taiwan
[8]Center of Applied Nanomedicine, National Cheng Kung University, Tainan 701, Taiwan
[9]Department of Biomedical Imaging and Radiological Sciences, National Yang Ming Chiao Tung University, Taipei 112, Taiwan
[10]Department of Radiation Oncology, Kaohsiung Chang Gung Memorial Hospital, Kaohsiung 833, Taiwan
[11]Institute for Radiological Research, Chang Gung University, Taoyuan, 333, Taiwan
[12]Department of Chemical Engineering, National Cheng Kung University, Tainan 701, Taiwan

* Corresponding author e-mail: wpli@kmu.edu.tw, chiralsu@gmail.com, hktian@gs.ncku.edu.tw, csyeh@mail.ncku.edu.tw

Additional descriptions

Calculation of the photothermal conversion efficiency of CoFe Prussian Blue (CFPB) nanoframes under 808 nm laser irradiation.

The photothermal conversion efficiency (η) of CFPB nanoframes was determined as follows.

The η can be calculated by the equation below.

$$\eta = \frac{hS(T_{max} - T_{surr}) - Q_S}{I(1-10^{-A})} \quad (1)$$

Where h is the heat transfer coefficient. S is the surface area of the well. T is the temperature of the solution. Q_s is the surrounding heat associated with the light absorbance by solvent and well. I is the power of the laser. A is the absorbance of nanomaterials at 808 nm.

The equation (1) is defined based on the total energy balance of the system:

$$\sum_i m_i Cp_i \frac{dT}{dt} = Q_{NPs} + Q_s - Q_{loss} \quad (2)$$

The m and C_p are the mass and heat capacity of the solvent, respectively.

The Q_{NPs} is the photothermal energy input from CFPB nanoframes.
And Q_{NPs} can be expressed as equation (3):

$$Q_{NPs} = I(1-10^{-A})\eta \quad (3)$$

The Q_s can be measured by irradiating the solvent (pure water without the nanomaterials). The values of Q_S are 0.0005 (J K s^{-1}) under laser irradiation at 808 nm.

The Q_{loss} is the thermal energy lost to the surroundings, and it can be determined by the equation below:

$$Q_{loss} = hS(T_{max} - T_{surr}) \quad (4)$$

Where hS can be defined to equation (5) and τ is a sample system time constant.

$$hS = \frac{mCp}{\tau} \quad (5)$$

The τ_s can be calculated from the cooling period (after 900 sec.) vs ln(θ).
The θ is the dimensionless driving force temperature and can be defined to:

$$\theta = \frac{(T - T_{surr})}{(T_{max} - T_{surr})} \quad (6)$$

The $\tau_{s\text{-}CFPB}$ was calculated as 217.4 (sec.).
And then, we can obtain hS of CFPB nanoframes, which is 0.002 W.

The equation (7) is defined based on the equation (2) under a condition of total energy balance:

$$Q_{NPs} + Q_s = hS(T_{max} - T_{surr}) \quad (7)$$

Thus, we can get the equation (1) according to equation (3) and equation (7).

Based on our laser irradiation system, the below parameters can be obtained by experiments. I is 0.288 W. (T_{max}-T_{surr}) can be measured as 19.3 (ºC) by irradiation of the CFPB nanoframes in 10 ppm of iron ion concentration for 900 s. A value of CFPB nanoframes is 1.206. Finally, we can calculate the η for CFPB nanoframes as 13.6 %.

Kinetics of the proton-induced metal replacement reaction in different PBAs.

Preparation of the MnFe and NiFe PB (respectively simplified as MFPB and NFPB) yields nanocubes with edge lengths of 200 and 166 nm, respectively (Supplementary Fig. 18a). The MFPB nanocubes exhibit extremely rough surfaces, whereas the NFPB nanocubes have smooth faces with sharp edges and corners. The coarse surface of the MFPB nanocube has numerous defects. Irregular NPs, which we consider fragments derived through the dissolution of the MFPB nanocubes, form aggregates under 24-h etching by 0.01 M HCl, indicating extreme corrosion. Although no well-defined frame-like structure is generated, these aggregates display NIR absorption, suggesting the occurrence of the proton-induced metal replacement reaction (Supplementary Fig. 18b).

The smooth-surfaced NFPB nanocubes are relatively resistant to acid corrosion, corresponding to a slow etching process. They require considerably more time to generate frame-like structures than the dose from MFPB nanocubes. The TEM images in Supplementary Fig. 18a display the products of 1-, 5-, and 15-day HCl treatment (0.01 M). Concave structures are obtained through the first day. A residual nanocube residing in a nanoframe, as well as some other nanoframes, is noted after the reaction proceeds for 5 days. When the reaction is extended to 15 days, the frame-like structures become dominant with solid NPs (as by-products) observed occasionally. In the optical spectra, an increase in NIR absorption following the proton-induced metal replacement reaction is observable (Supplementary Fig. 18c).

Additional methods

Synthesis of manganese-iron Prussian blue (MFPB) nanocubes. 0.05 g of potassium hexacyanoferrate(III) was dissolved in 30 mL of H_2O. 0.0733 g of manganese(II) acetate and 0.294 g of trisodium citrate dihydrate were dissolved in another 20 mL of H_2O. Then, both solutions were mixed together and stirred in an ice bath. After 15 min, the precipitate was collected with centrifugation at 8700 g for 5 min and washed with 90% ethanol for three times. The precipitates were collected through centrifugation at 8900 g for 5 min and washed with 90%/10% of ethanol/deionized water. We repeatedly washed and centrifuged the MFPB nanocubes at least three times. Finally, the MFPB nanocubes were dispersed in deionized water for future use.

Acid-etching reaction of MFPB nanocube. MFPB nanocubes (400 ppm in Fe ion concentration) were dissolved in 2 mL of H_2O and then mixed with 14 mL of 0.01M HCl solution. Next, the solution was stirred and heated in an oil bath at 90°C for different times (5, 10, 15, 20, 40 min, and 1 day). After the acid-etching reaction, That solution was collected, and then centrifuged at 7800 g for 5 min. The supernatants were removed and the precipitates were redispersed into 90%/10% of ethanol/deionized water. The processes of washing and centrifuging were repeated at least three times.

Synthesis of nickel-iron Prussian blue (NFPB) nanocubes. 0.05 g of potassium hexacyanoferrate(III) was dissolved in 30 mL of H_2O. 0.1054 g of nickel(II) acetate tetrahydrate and 0.147 g of trisodium citrate dihydrate were dissolved in another 20 mL of H_2O. Then, both solutions were mixed together and stirred in an ice bath. After 24 h, the precipitation was collected through centrifugation at 8700 g for 5 min, and then washed with 50%/50% of ethanol/deionized water (volume/volume). We repeatedly washed and centrifuged the NFPB nanocubes at least three times. Finally, the NFPB nanocubes were dispersed in deionized water for future use.

Acid-etching reaction of NFPB nanocubes. NFPB nanocubes (400 ppm in Fe ion concentration) were dissolved in 2 mL of H_2O and then mixed with 14 mL of 0.01M HCl solution. Next, the solution was stirred and heated in an oil bath at 90°C for different times (1, 5, 12, and 15 days). After the acid-etching reaction, the solution was collected, and then centrifuged at 8700 g for 5 min. The supernatants were removed and the precipitates were redispersed into 50%/50% of ethanol/deionized water (volume/volume). The processes of washing and centrifuging were repeated at least three times.

Synthesis of Prussian blue (PB) nanocubes. 6.0 g of polyvinylpyrrolidone (PVP) was dissolved in 60 mL 0.1M of HCl solution. The solution was stirred until the PVP powder completed dissolved. And then, 0.05 g of potassium hexacyanoferrate(III) was added to the solution. The solution was put into the autoclave and then heated at 80°C for 1 h. The solution was collected and then centrifuged at 8700 g for 5 min. The supernatants were removed and the precipitates were redispersed into ethanol-water co-solvent. We repeatedly washed and centrifuged the PB nanocubes at least three times. Finally, the PB nanocubes were dispersed in deionized water for future use.

Acid-etching reaction of PB nanocubes. PB nanocubes (400 ppm in Fe ion concentration) were dissolved in 2 mL of H_2O and then mixed with 14 mL of 0.01M HCl solution. Next, the solution was stirred and heated in an oil bath at 90°C for 1 day.

After the acid-etching reaction, the solution was collected, and then centrifuged at 7800 g for 5 min. The supernatants were removed and the precipitates were redispersed into 50%/50% of ethanol/deionized water (volume/volume). The processes of washing and centrifuging were repeated at least three times.

Supplementary Table. 1 Measured raw data of Fe and Co elements in different etching reaction times for CFPB nanoparticles. All data were obtained from atomic absorption spectroscopic (AAS) measurements in triplicate.

	Fe (ppm)			Co (ppm)		
Time (hour)	Duplicate 1	Duplicate 2	Duplicate 3	Duplicate 1	Duplicate 2	Duplicate 3
0	1.66	1.74	1.63	2.89	2.62	2.54
0.5	1.16	1.05	1.18	1.22	1.41	1.39
1	1.31	1.11	1.45	1.45	1.18	1.15
3	1.11	1.21	1.38	1.35	0.81	0.80
16	0.78	0.80	0.43	0.62	0.23	0.21
24	0.63	0.70	0.56	0.44	0.15	0.13

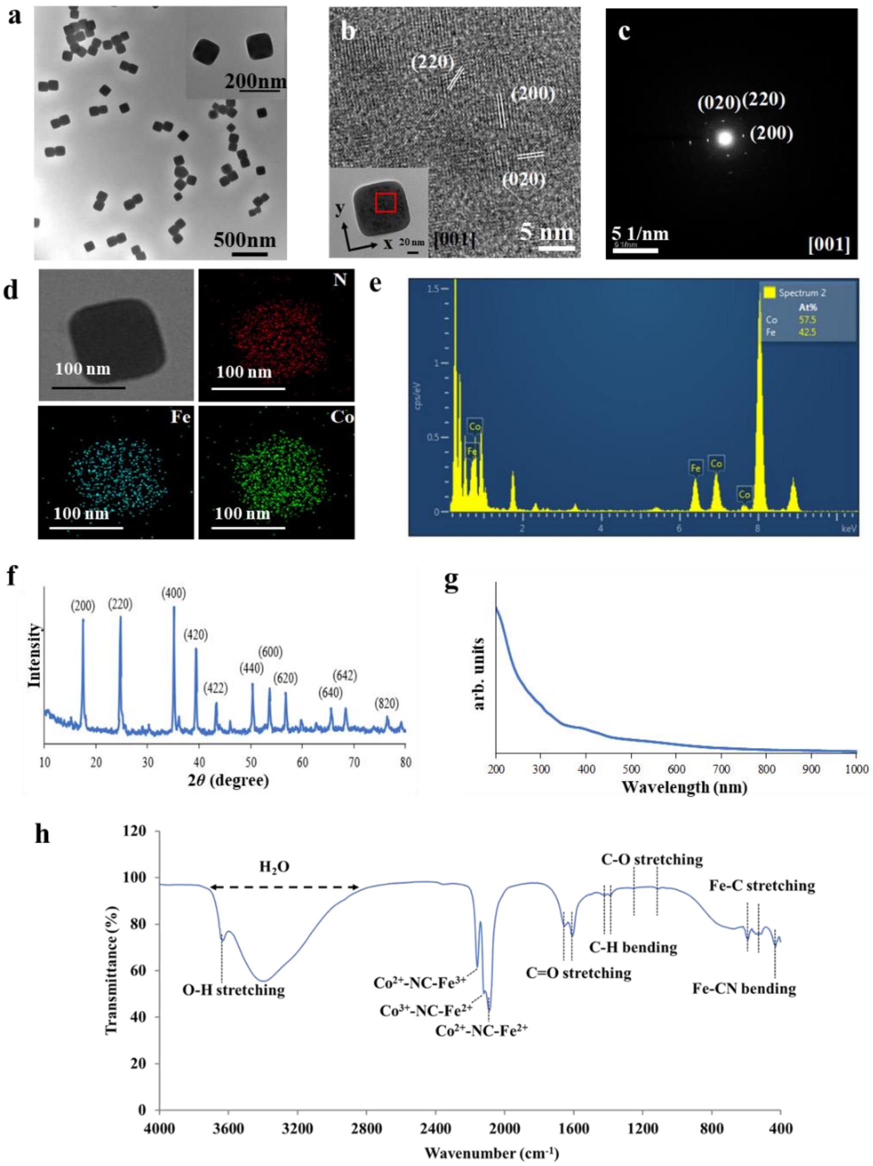

Supplementary Fig. 1 Basic characteristics of CFPB nanocubes. a, Transmission electron microscopy (TEM) image of CFPB nanocubes. Inset: A high-magnification image **b,c,** Crystallographic and electron diffraction data obtained through high-resolution TEM, indicating the arrangements of (200), (020), and (220) planes. Inset in **b,** The corresponding area for lattice observation (red box). **d,** Homogeneous distribution of N, Co, and Fe in a CFPB nanocube under high-resolution TEM (mapping mode). **e,** Content ratios of Co and Fe were determined through EDX to be 57.5% and 42.5 %, respectively. **f,** XRD results indicating a well-defined CFPB signal (JCPDS cards 00-046-0907) with a face-centered cubic crystal structure. **g,** UV–Vis spectra of CFPB nanocubes demonstrating absorbance between 200 and 600 nm showing no characteristic absorbance beyond 700 nm in the NIR region. **h,** FTIR spectrum presenting vibration peaks of CFPB nanocubes with evidence of Co^{2+}-N≡C-Fe^{3+}, Co^{3+}-N≡C-Fe^{2+} and Co^{2+}-N≡C-Fe^{2+} signals. A vibration signal corresponding to a carbonyl group (C=O), observed at approximately 1700 cm^{-1}, attributed to the citrate capping agent on the CFPB nanocube surfaces. (One representative data was shown from three independently repeated experiments)

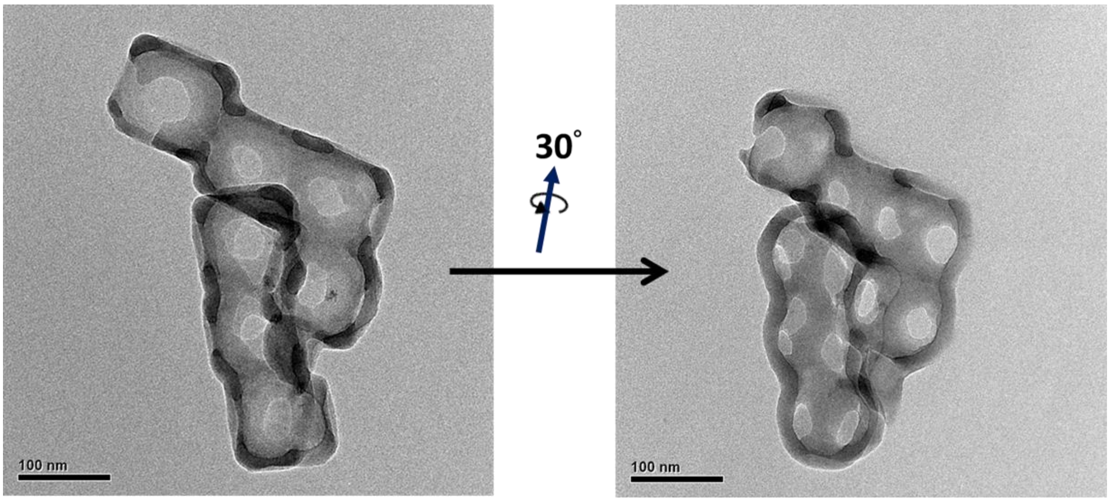

Supplementary Fig. 2 High-resolution TEM images of CFPB nanoframes tilted at 30°. (One representative data was shown from three independently repeated experiments)

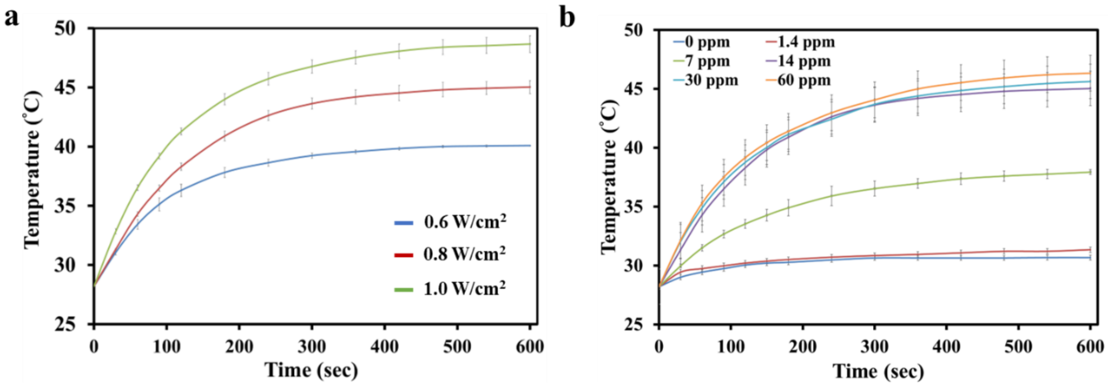

Supplementary Fig. 3 Heating performance of CFPB nanoframes in H_2O. Heating performance was evaluated under exposure to an 808-nm laser diode at various intensities with **a,** a fixed 14 ppm cobalt concentration and **b,** under various nanoframe concentrations with exposure to an 808-nm laser diode at 0.8 W/cm^2. All data were obtained in triplicate. (n=3, The error bars represented mean ± SD. Source data are provided as a Source Data file.)

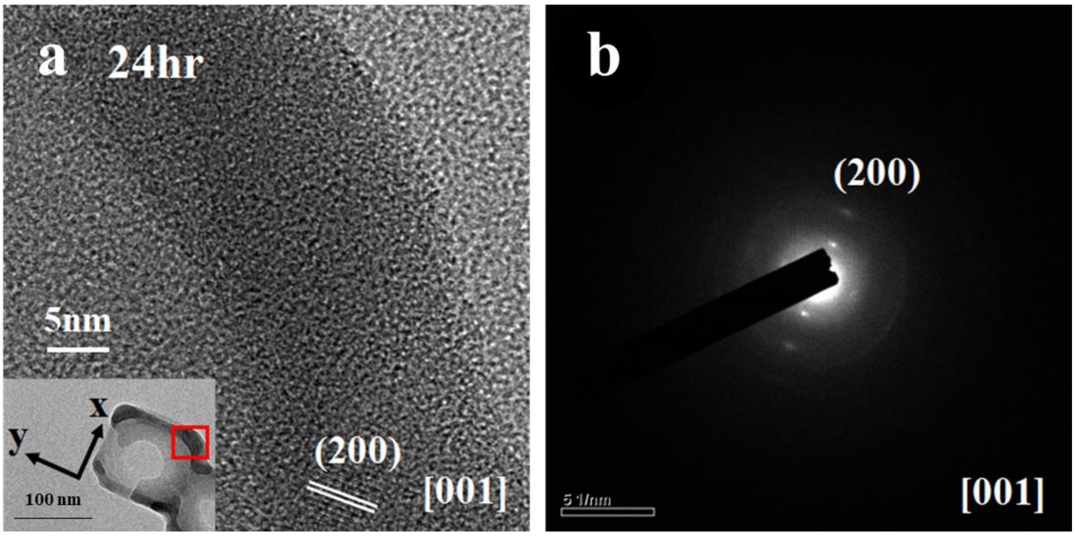

Supplementary Fig. 4 High-resolution TEM images and electron diffraction images of a nanoframe after 24 h of reaction. a, Crystal arrangement of the (200) plane at the edge region of a CFPB nanoframe. **b,** Diffraction points of the (200) plane obtained from a CFPB nanoframe. The red box corresponds to the selected area of the CFPB nanoframe for analysis. (One representative data was shown from three independently repeated experiments)

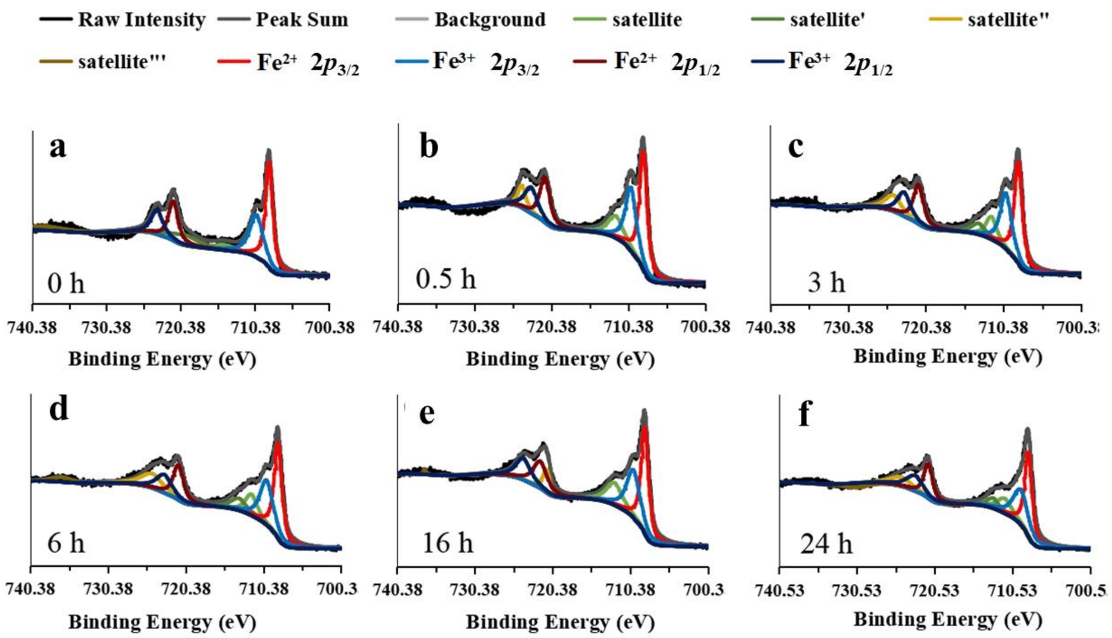

Supplementary Fig. 5 XPS analysis of iron oxidation states in nanostructures. The spectra show Fe^{2+} and Fe^{3+} signals, monitored as a function of the etching duration, with CFPB nanocubes as the starting material. **a,** 0 h. **b,** 0.5 h. **c,** 3 h. **d,** 6 h. **e,** 16 h. **f,** 24 h. The binding energies of Fe^{3+} in $2p_{3/2}$ and $2p_{1/2}$ orbitals were 709.4 and 723.5, respectively. The binding energies of Fe^{2+} in $2p_{3/2}$ and $2p_{1/2}$ orbitals were 708.4 and 721.3, respectively. Each raw peak can be split into specific oxidation states. The integral area of each peak is calculated as the Fe^{2+} or Fe^{3+} content in each reaction stage.

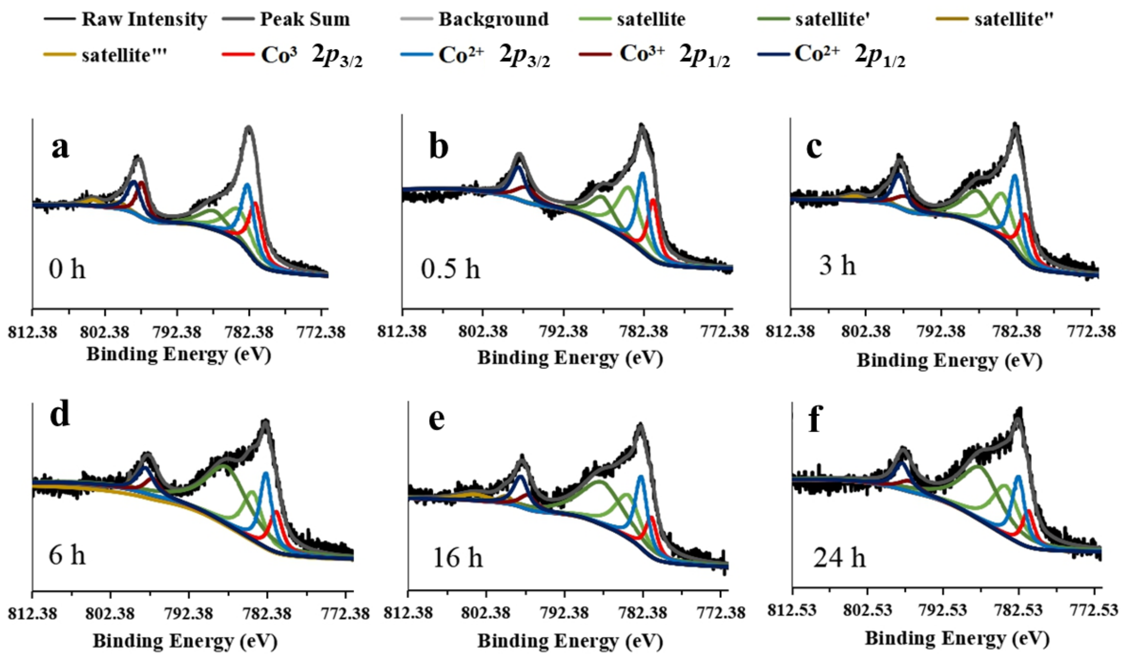

Supplementary Fig. 6 XPS analysis of cobalt oxidation states in nanostructures. The spectra show Co^{2+} and Co^{3+} signals, monitored as a function of the etching duration, with CFPB nanocubes as the starting material. **a,** 0 h. **b,** 0.5 h. **c,** 3 h. **d,** 6 h. **e,** 16 h. **f,** 24 h. The binding energies of Co^{3+} in $2p_{3/2}$ and $2p_{1/2}$ orbitals were 781.2 and 797.0, respectively. The binding energies of Co^{2+} in $2p_{3/2}$ and $2p_{1/2}$ orbitals were 782.5 and 797.9, respectively. Each raw peak can be split into specific oxidation states. The integral area of each peak was calculated as the Co^{2+} or Co^{3+} content in each reaction stage.

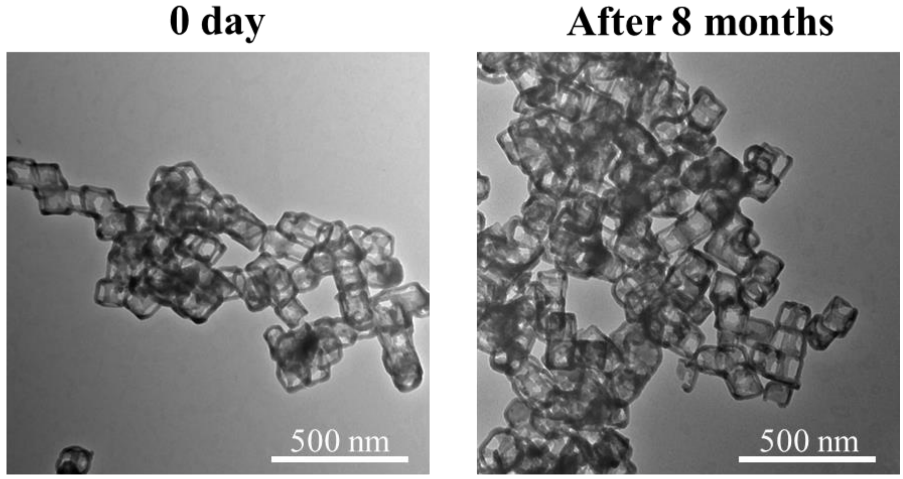

Supplementary Fig. 7 Stability test of the CFPB nanoframes dispersed in water. TEM images of the nanoframes before and after storage for 8 months. (One representative data was shown from three independently repeated experiments)

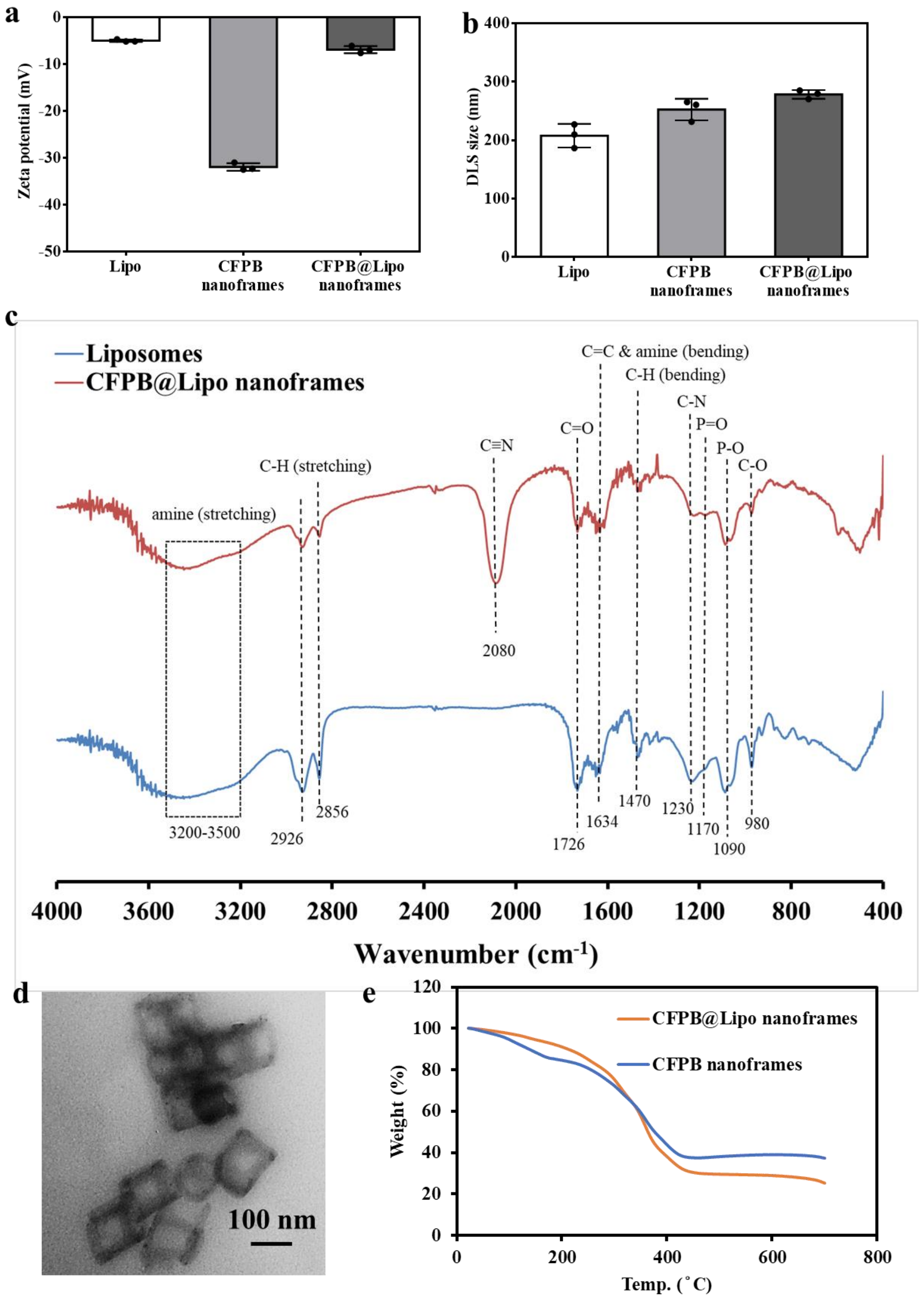

Supplementary Fig. 8 Evaluation of the physical properties before and after functionalization of CFPB nanoframes. a, The zeta potentials of Lipo, CFPB nanoframes and CFPB@Lipo nanoframes. **b,** The hydrodynamic diameters of Lipo, CFPB nanoframes and CFPB@Lipo nanoframes. **c,** FTIR spectra presenting vibration peaks of CFPB@Lipo nanoframes and liposomes. **d,** TEM images after Lipo coating. Scale bar units is 100 nm **e,** TGA analysis for CFPB@Lipo nanoframes and CFPB nanoframes. All data were obtained in triplicate (n=3, The error bars represented mean ± SD. Source data are provided as a Source Data file.) (c,d, One representative data was shown from three independently repeated experiments)

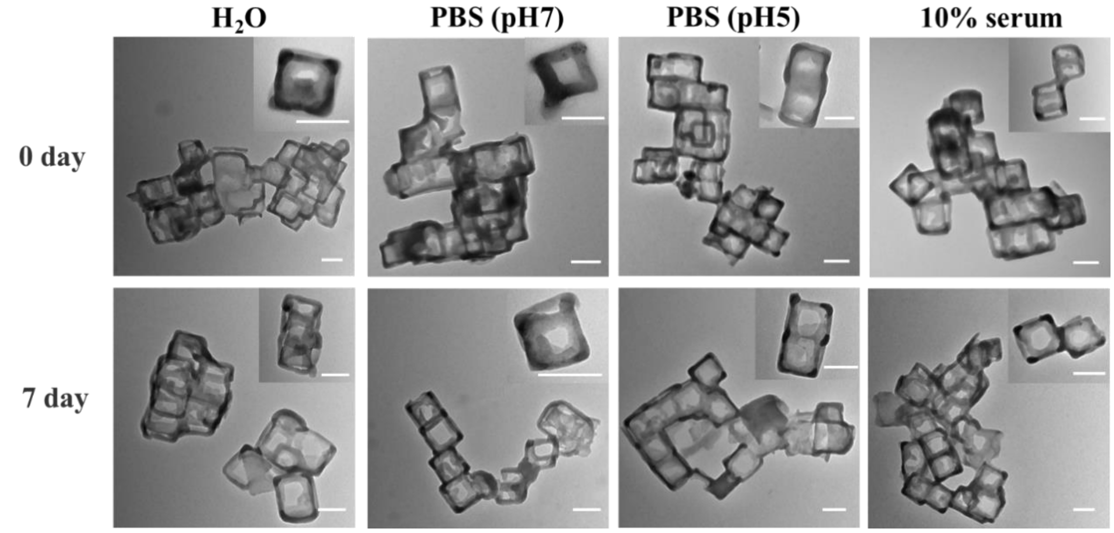

Supplementary Fig. 9 Stability performance of CFPB@Lipo nanoframes in various solutions at 37 °C under water, PBS (pH 7), PBS (pH 5), and serum. TEM images reveal that the nanoframes remain intact structures across all conditions over 7 days of incubation. All scale bar units are 100 nm. (One representative data was shown from three independently repeated experiments)

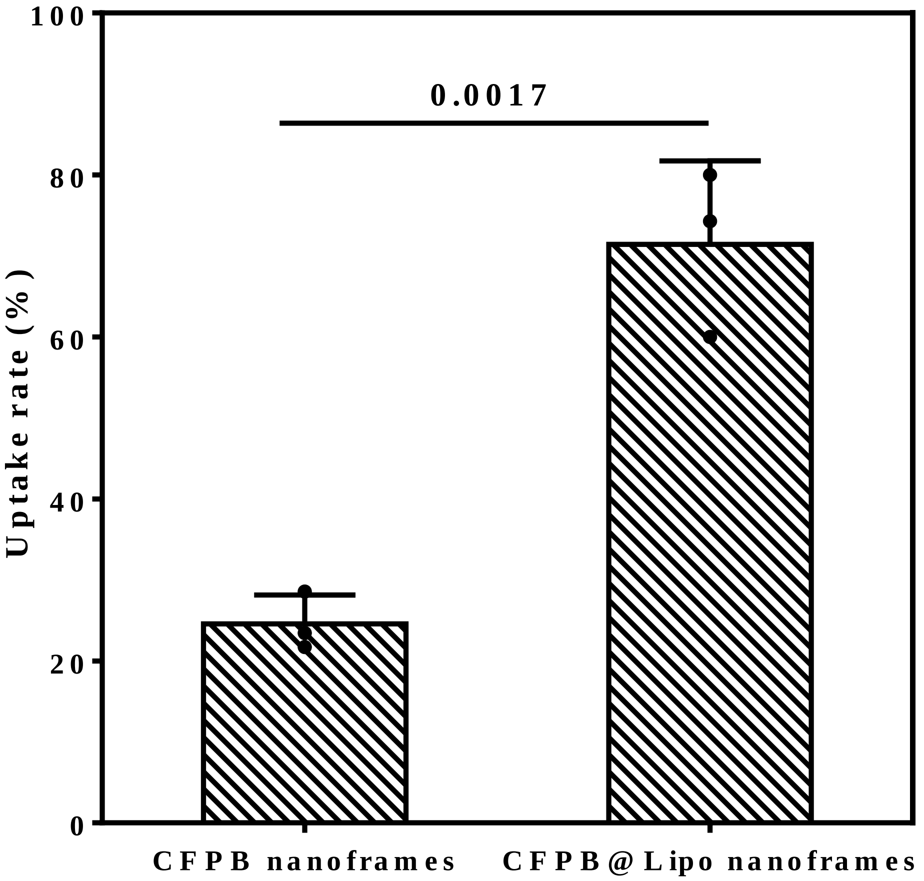

Supplementary Fig. 10 Cellular uptake of CFPB and CFPB@Lipo nanoframes. All data were obtained in triplicate (n=3, The error bars represented mean ± SD. p-values were calculated by one-way ANOVA. Source data are provided as a Source Data file.)

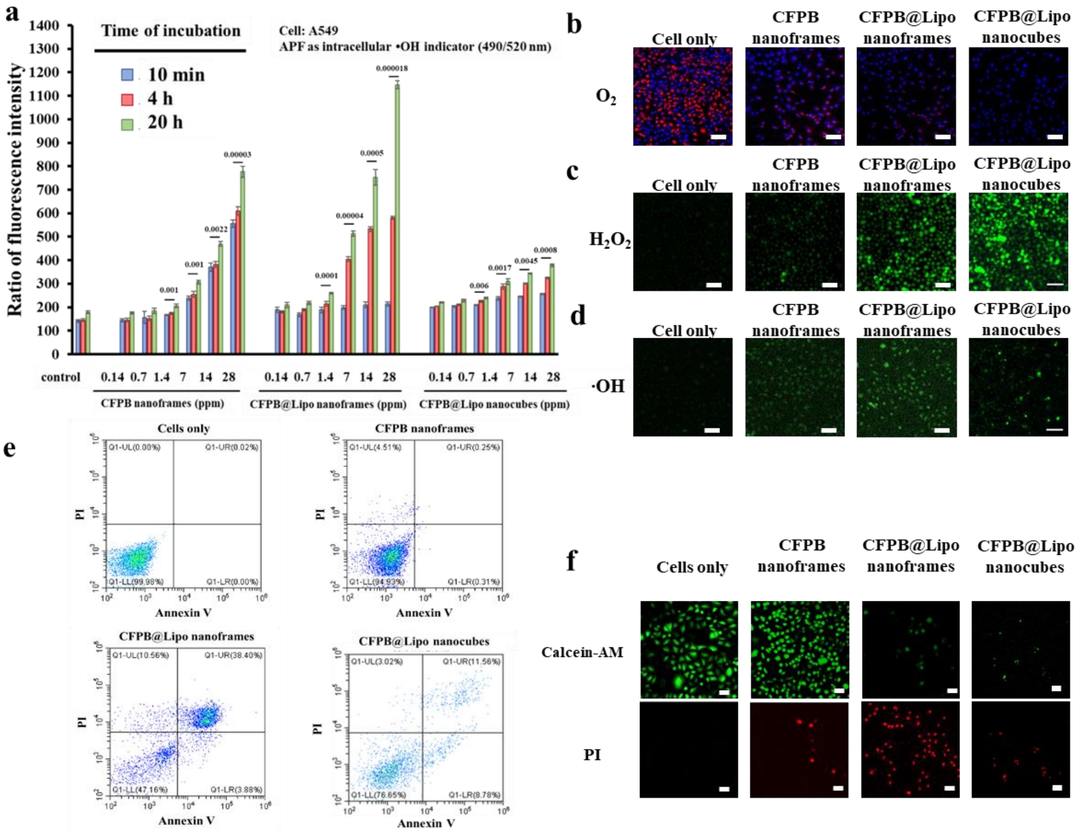

Supplementary Fig. 11 ***In vitro* A549 cells studies of CDT and cytotoxicity studies.** **a,** Fluorescence intensity of APF representing the level of ·OH generated from the control group (buffer only), CFPB nanoframes, CFPB@Lipo nanoframes and CFPB@Lipo nanocubes under various concentrations over 10 min, 4 h, and 20 h. (n=3, The error bars represented mean ± SD, p-values were calculated by one-way ANOVA) **b,** A549 cells treated with [Ru(dpp)$_3$]Cl$_2$ dye (as the control group), [Ru(dpp)$_3$]Cl$_2$ dye + CFPB nanoframes, [Ru(dpp)$_3$]Cl$_2$ dye + CFPB@Lipo nanoframes and [Ru(dpp)$_3$]Cl$_2$ dye + CFPB@Lipo nanocubes under a 30-min incubation to monitor O_2 generation . **c,** A549 cells treated with DCFH-DA dye (as the control group), DCFH-DA dye + CFPB nanoframes, DCFH-DA dye + CFPB@Lipo nanoframes, and DCFH-DA dye + CFPB@Lipo nanocubes under a 30-min incubation to monitor H_2O_2 generation (green emissions). **d,** A549 cells treated with APF dye (as the control group), APF dye + CFPB nanoframes, APF dye + CFPB@Lipo nanoframes, and APF dye + CFPB@Lipo nanocubes under a 30-min incubation to monitor H_2O_2 generation (green emissions). **e,** Flow cytometry analysis of A549 cancer cells with and without CFPB nanoframes, CFPB@Lipo nanoframes, and CFPB@Lipo nanocubes. The cells incubated with CFPB@Lipo nanoframes for 24 h have a relatively higher late apoptotic ratio (38.40%) compared to other groups (cell only: 0.02%, CFPB nanoframes: 0.25% and CFPB@Lipo nanocubes: 11.56%). **f,** Live and dead staining for CFPB nanoframes, CFPB@Lipo nanoframes, and CFPB@Lipo nanocubes. Significant damage in cells is seen in CFPB@Lipo nanoframes. All data were obtained in triplicate. scale bar: 200 µm

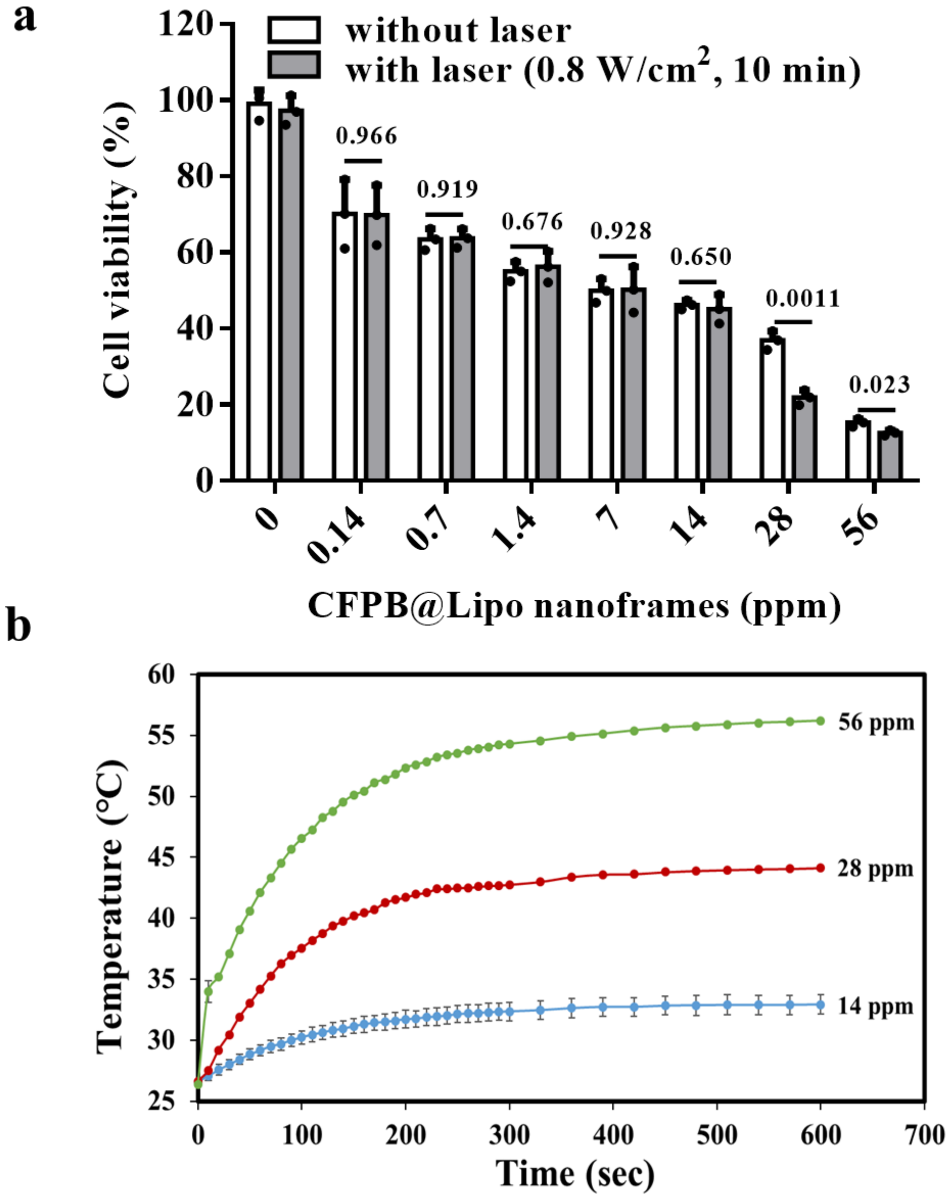

Supplementary Fig. 12 *In vitro* studies of PTT. a, Cells viability treated with various concentrations of CFPB@Lipo nanoframes with or without 10-min exposure to a 808-nm laser diode at 0.8 W/cm^2. **b,** Heating performance of CFPB@Lipo nanoframes in a cell culture system as a function of the duration of exposure to a 808-nm laser diode at 0.8 W/cm^2. All data were obtained in triplicate (n=3, The error bars represented mean ± SD, p-values were calculated by one-way ANOVA($*P < 0.05$, $***P < 0.001$). Source data are provided as a Source Data file.).

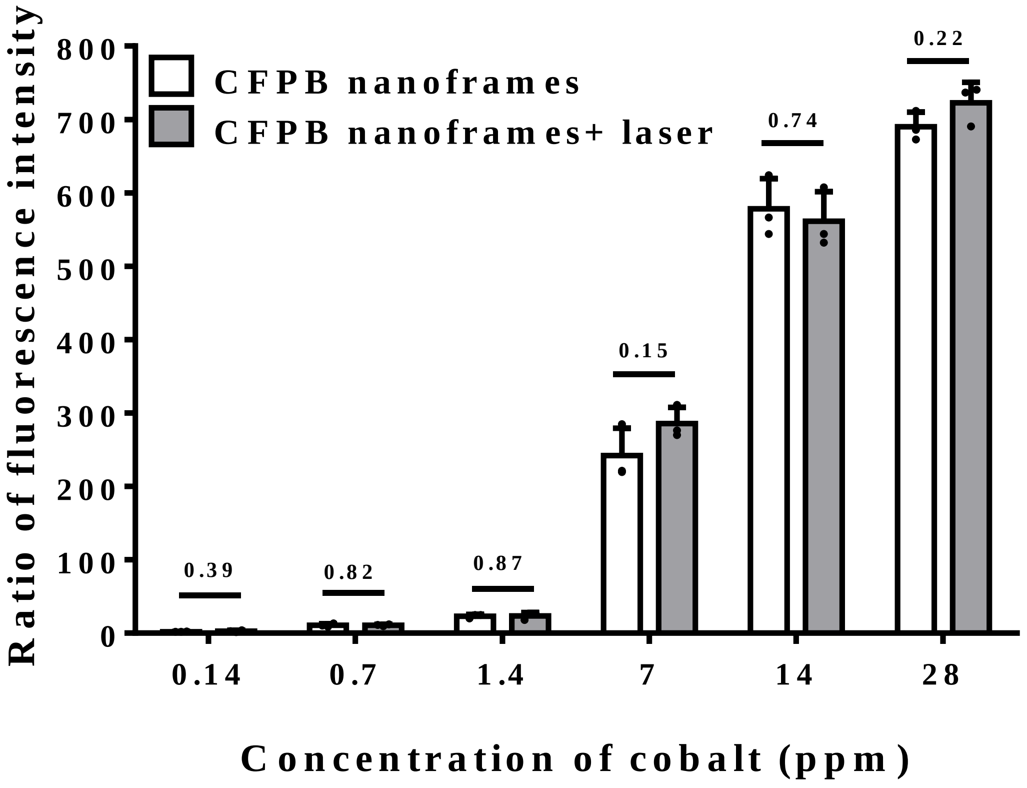

Supplementary Fig. 13 ·OH evaluation from CFPB nanoframes following with or without 10-min exposure to an 808-nm laser diode at 0.8 W/cm^2 under various concentrations. No significant difference is seen between with and without laser exposure. All data were obtained in triplicate (n=3, The error bars represented mean ± SD, p-values were calculated by one-way ANOVA (ns: no significance). Source data are provided as a Source Data file.).

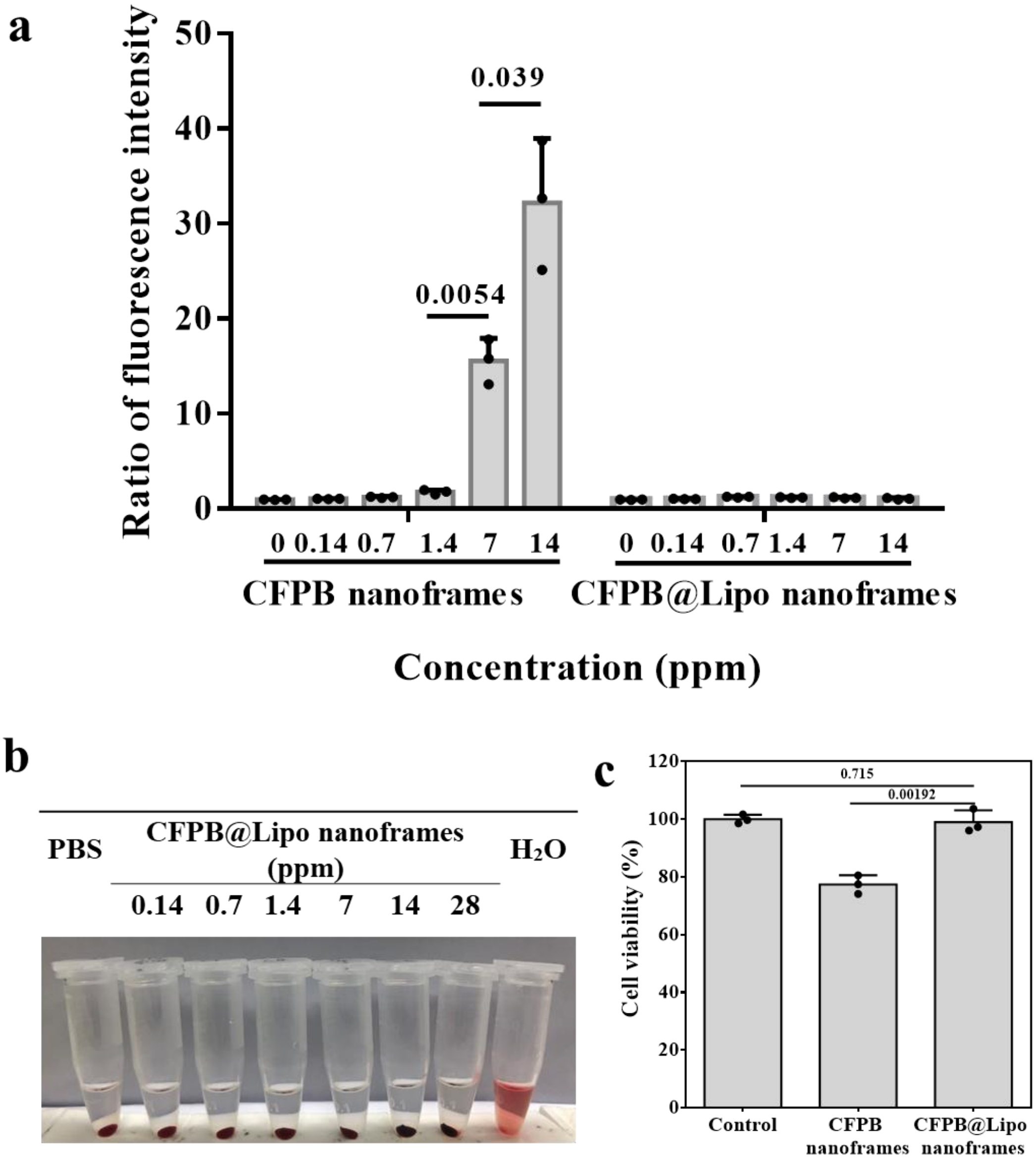

Supplementary Fig. 14 ***In vitro*** **studies. a,** ·OH observation in blood from CFPB and CFPB@Lipo nanoframes. **b,** Analysis of hemolysis in blood containing 2% red blood cells from CFPB@Lipo nanoframes at 1 h incubation. Negative and positive controls were conducted by immersing red blood cells in phosphate-buffered saline (PBS) and water, respectively. One representative data was shown from three independently repeated experiments. **c,** Cytotoxicity analysis of vascular endothelial cells following with CFPB and CFPB@Lipo nanoframes individually at 1 h incubation. The cells survival rate drops apparently in CFPB nanoframes. All data were obtained in triplicate (n=3, The error bars represented mean ± SD, p-values were calculated by one-way ANOVA(*$P < 0.05$, ***$P < 0.001$). Source data are provided as a Source Data file.)

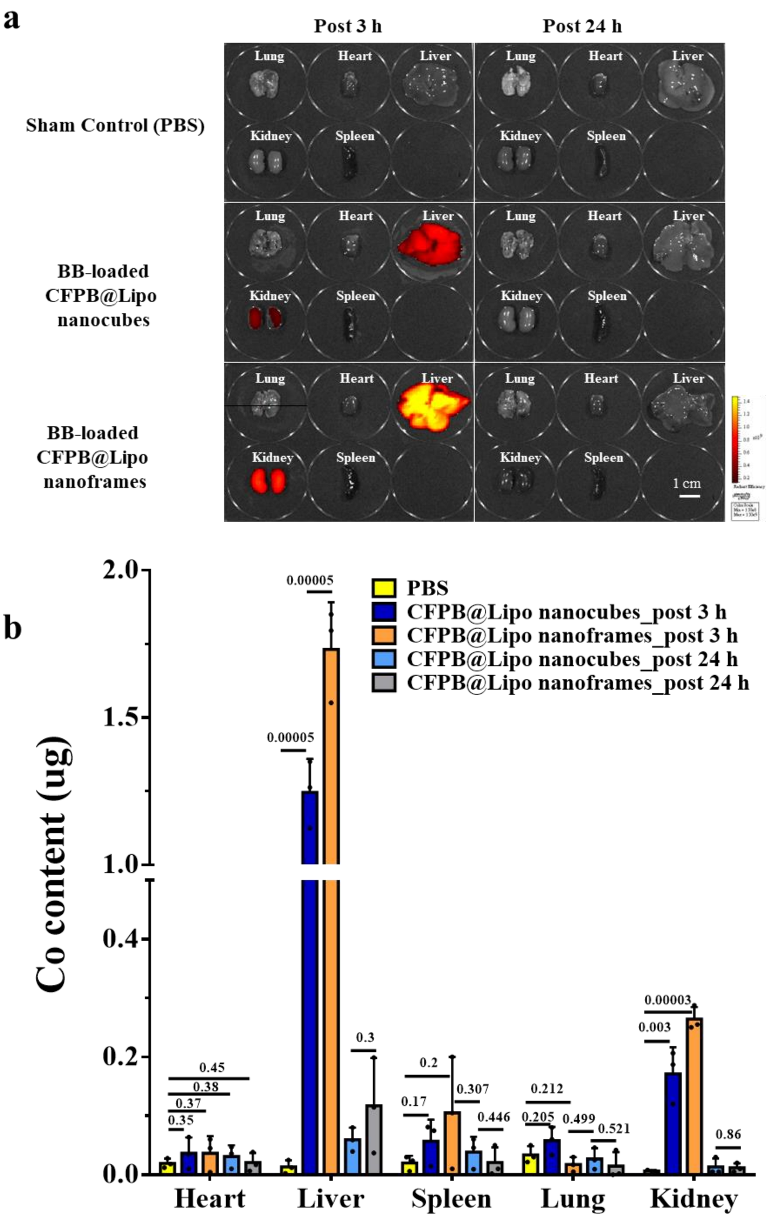

Supplementary Fig. 15 ***Ex vivo*** **fluorescence. a,** *Ex vivo* imaging was obtained from the dissected tissues at the post-injection 3 h and 24 h following the respective intravenous injection of RB-loaded CFPB@Lipo nanocubes and RB-loaded CFPB@Lipo nanoframes with the dosage of 100 ppm/mouse and the control set was injected with PBS. (ex.: 535 nm; em.: 580 nm; exposure time: auto; binning: 8; and f number: 2). The organs were harvested and performed *ex vivo* analysis by using IVIS imaging system (n= 3). The fluorescence signal intensities at 3 h post injection in the liver region were 2.81 × 10^{10} p/s/cm^2/sr for RB-loaded CFPB@Lipo nanocubes and 7.15 × 10^{10} p/s/cm^2/sr for RB-loaded CFPB@Lipo nanoframes. One representative data was shown from three independently repeated experiments. **b,** The biodistribution determined by Co concentration collected from CFPB@Lipo nanocubes and nanoframes for 3 and 24 h post intravenous injection. All data were obtained in triplicate (n=3, The error bars represented mean ± SD. p-values were calculated by one-way ANOVA(*P < 0.05, **P < 0.01, ***P < 0.001). Source data are provided as a Source Data file.)

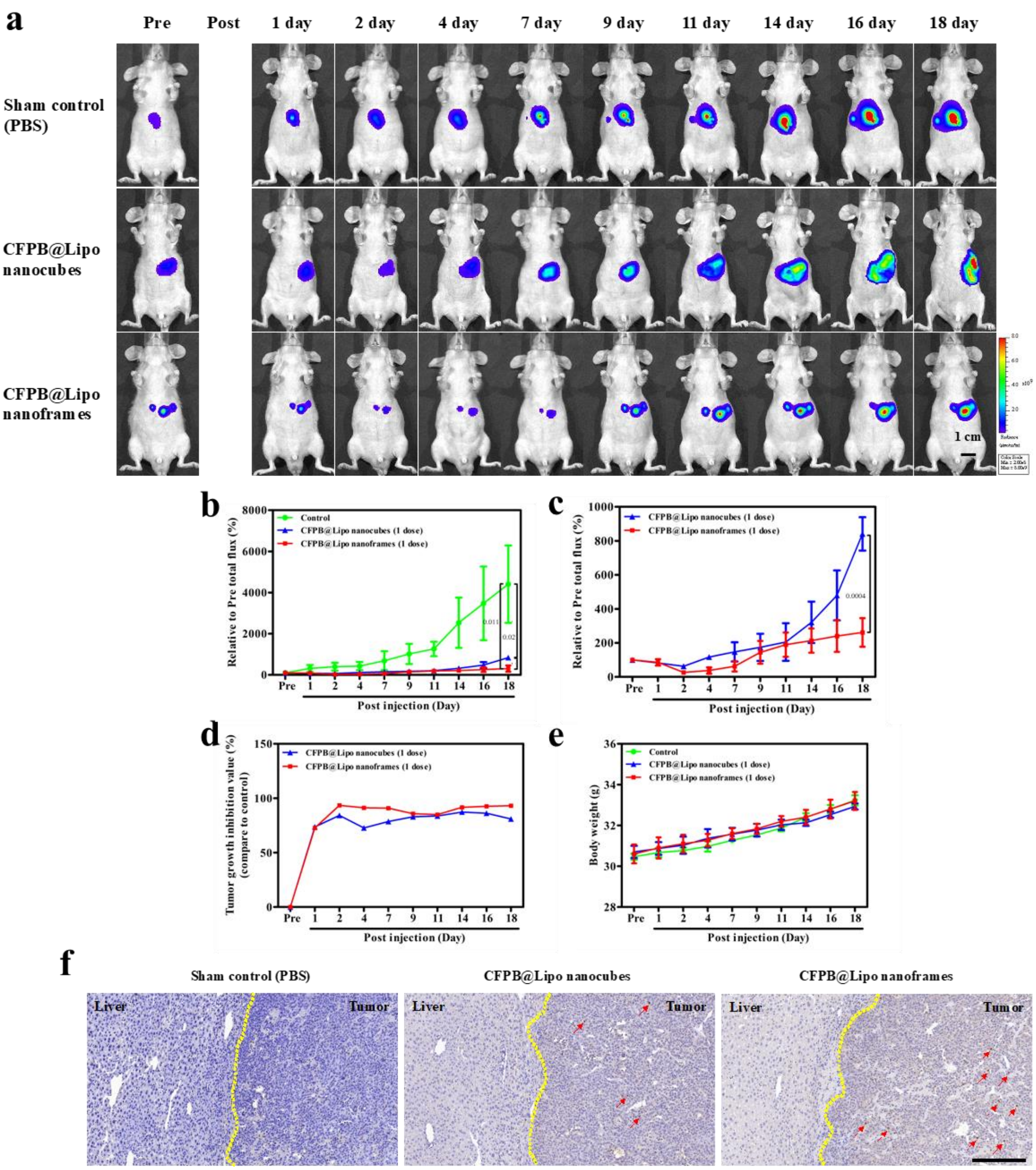

Supplementary Fig. 16 *In vivo* anti-tumor activity of the mice with Hep G2-Red-FLuc orthotopic tumors following single dose administration. a, The animal bioluminescence images from Hep G2-Red-FLuc cells were monitored using the IVIS imaging system. The mice were individually intravenously injected one dose (dosage: 100 ppm/mouse) of CFPB@Lipo nanocubes and CFPB@Lipo nanoframes and the control set was injected with PBS. **b,** The tumor growth profiles from the different treated groups (**P* < 0.05). **c,** The tumor growth profiles without control group (**P* < 0.05). **d,** Illustration of the tumor growth inhibition (TGI) rate. **e,** The variation of body weight from the different treated groups. **f,** The DNA damage of the tumor region in liver tissue. The expression of phospho-H2A.X was detected by IHC staining (red arrows: DNA damage markers within liver tumor cells; n= 3; scale bar: 200 μm). All data were obtained in triplicate (n=3, The error bars represented mean ± SD, p-values were calculated by one-way ANOVA(**P* < 0.05). (a, f, One representative data was shown from three independently repeated experiments)

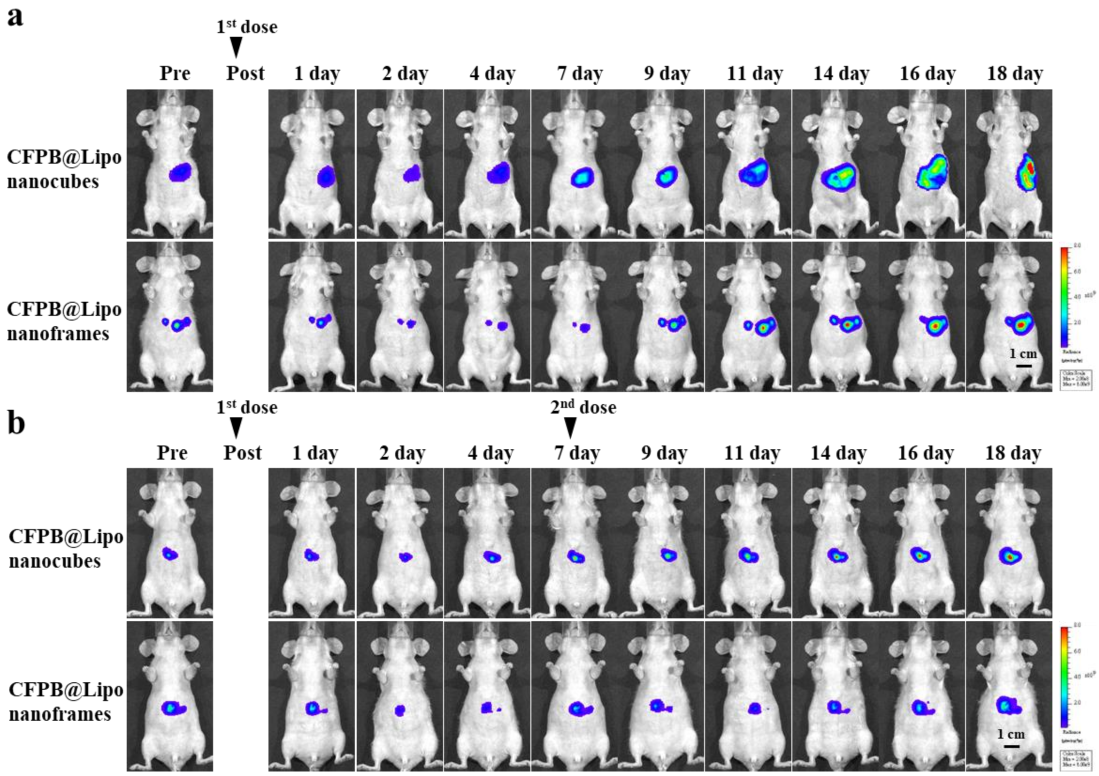

Supplementary Fig. 17 The comparison of animal bioluminescence images. The orthotopic tumors were treated with the **a,** single-dose and **b,** two-doses of CFPB@Lipo nanocubes and CFPB@Lipo nanoframes. (One representative data was shown from three independently repeated experiments)

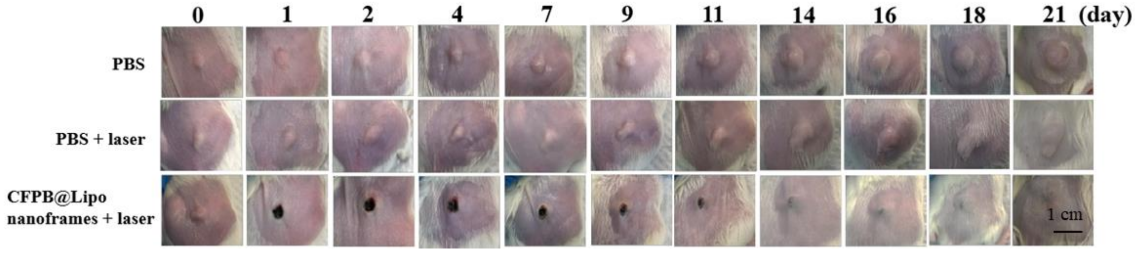

Supplementary Fig. 18 The photographs of superficial tumors on the mice. The mice were treated with PBS, PBS + laser, and CFPB@Lipo nanoframes + laser through intratumoral injection. The illumination for the local tumors was conducted for 10 min using an 808-nm laser diode at 0.8 W/cm^2. (One representative data was shown from three independently repeated experiments)

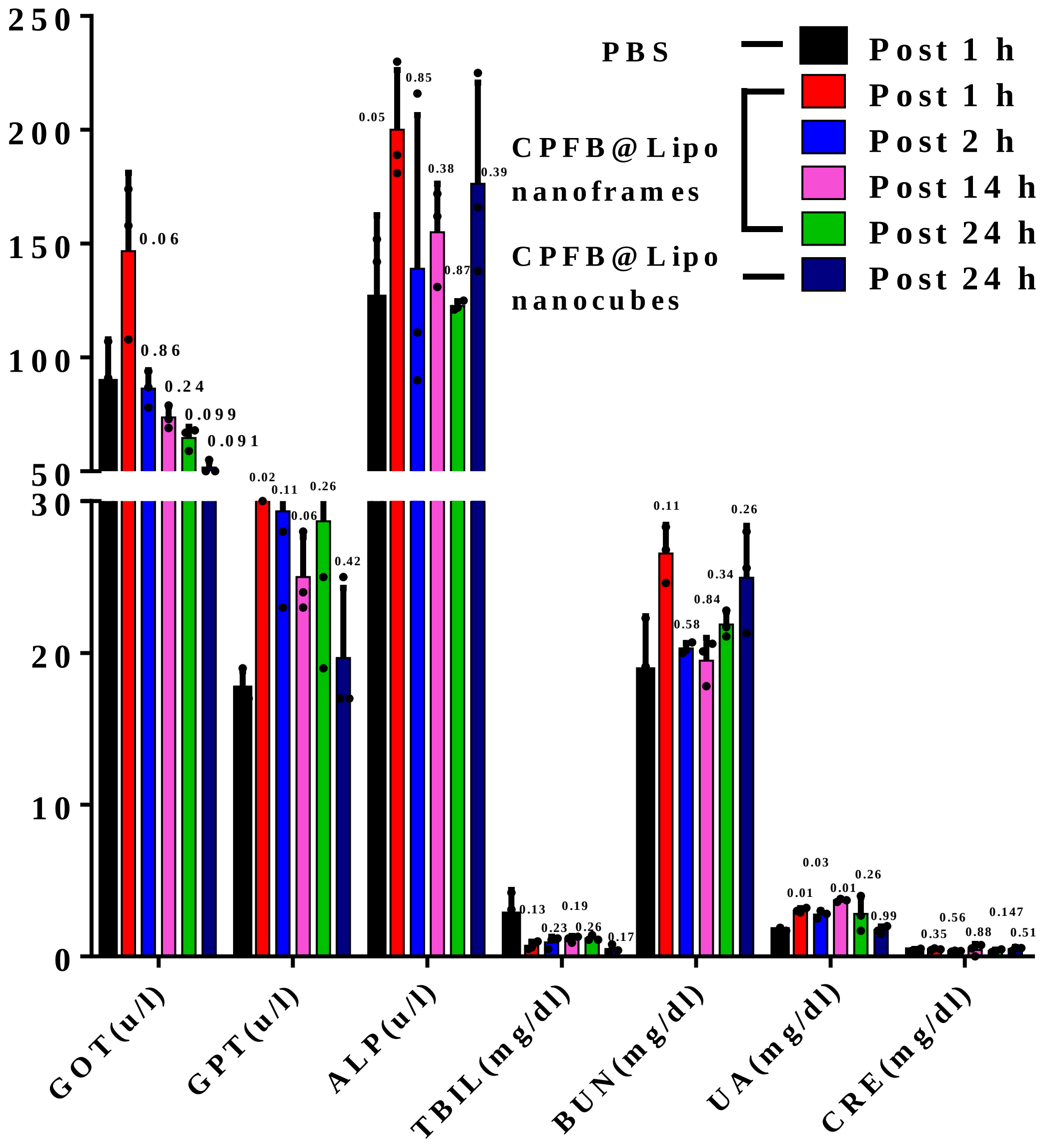

Supplementary Fig. 19 Blood biochemistry analysis. The blood was obtained from sacrificed mice after 1, 2, 14, and 24 h following intravenous injection of PBS, CFPB@Lipo nanoframes, and CFPB@Lipo nanocubes. (GOT: aspartate transaminase, GPT: alanine transaminase, ALP: alkaline phosphatase, TBIL: total bilirubin, BUN: blood urea nitrogen, UA: uric acid, and CRE: creatinine). All experiments were repeated three times (n=3). All data were obtained in triplicate (n=3, The error bars represented mean ± SD, p-values were calculated by one-way ANOVA(ns: no significance). Source data are provided as a Source Data file.)

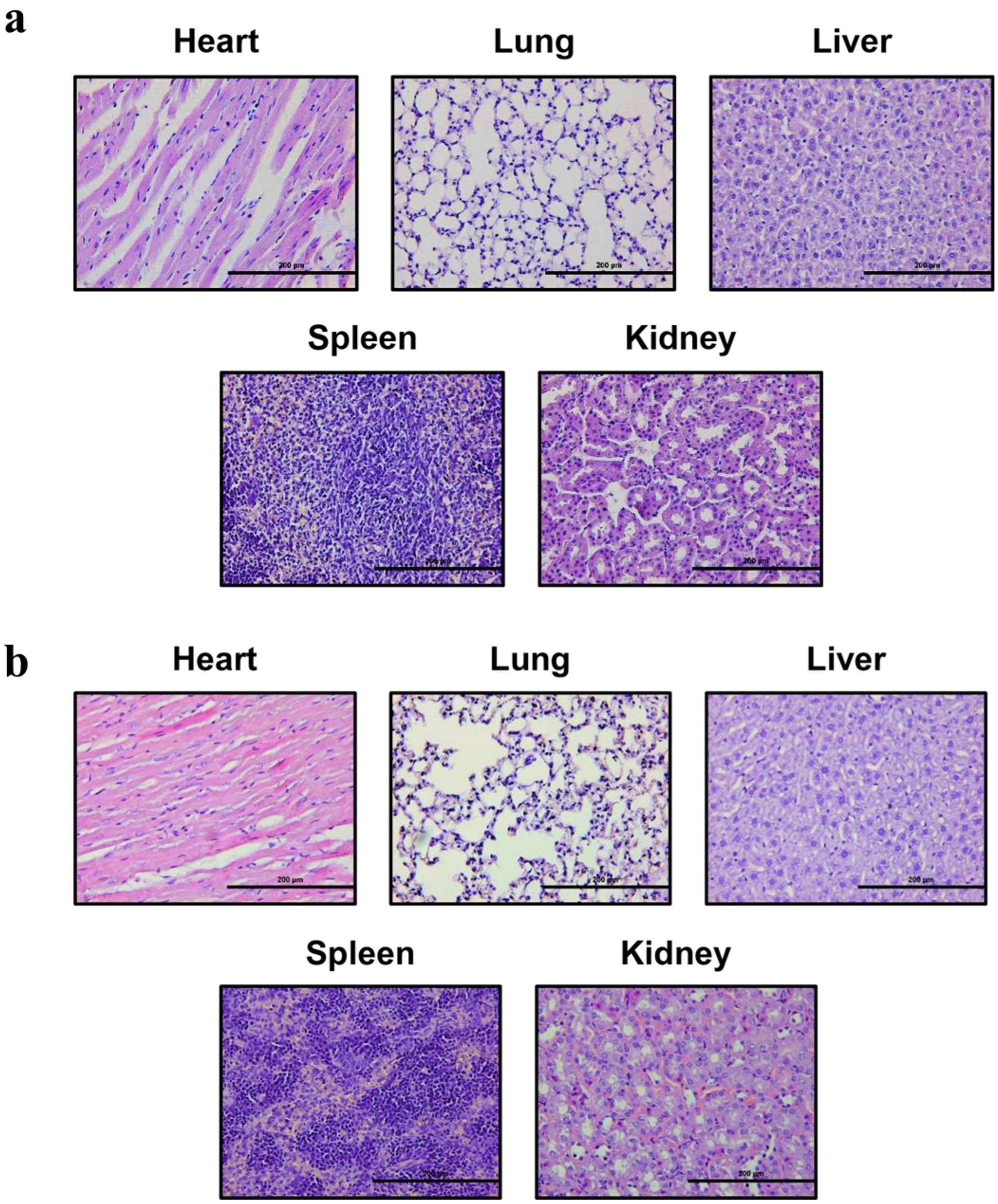

Supplementary Fig. 20 Histomorphological analysis. The histological morphology of heart, lung, spleen, liver and kidney from mice administering **a,** CFPB@Lipo nanoframes and **b,** CFPB@Lipo nanocubes after 24h post-treatment by H&E staining (Scale bar, 200 μm). (One representative data was shown from three independently repeated experiments)

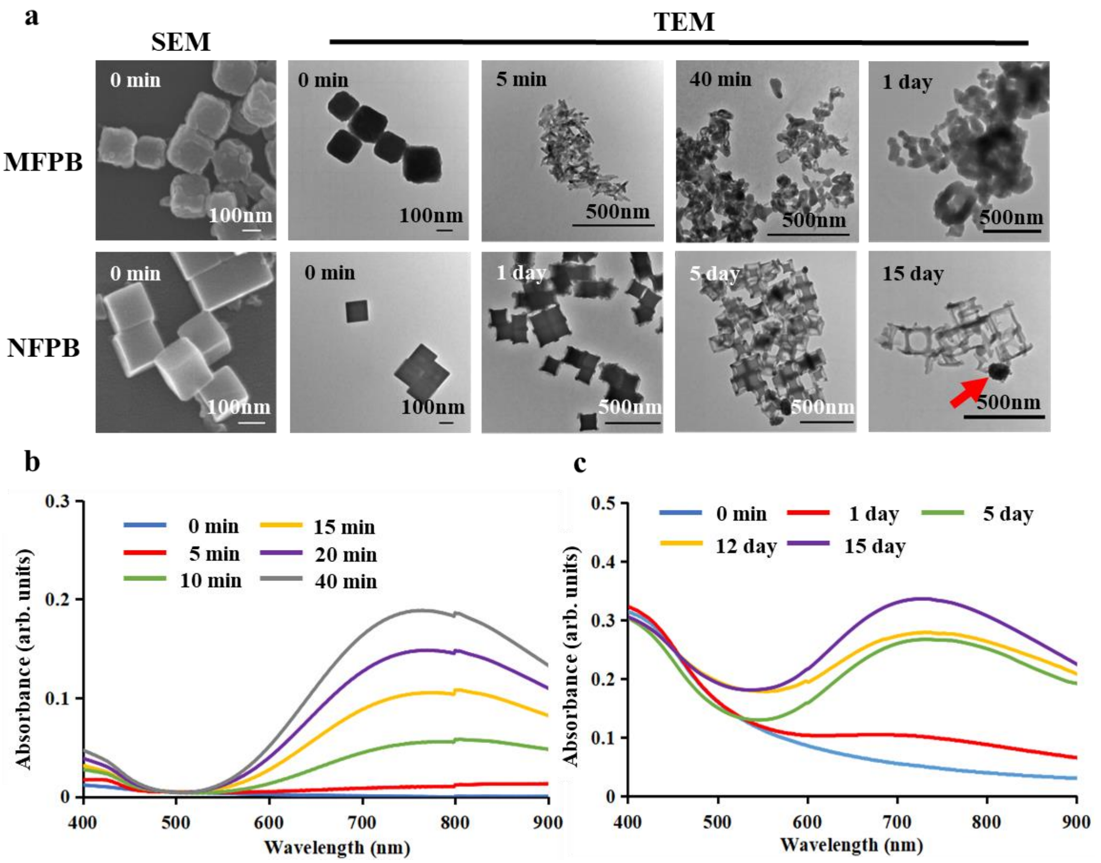

Supplementary Fig. 21 Evaluation of MFPB and NFPB following acid etching. a, The electron microscopy images of MFPB and NFPB nanostructures before and after the acid etching process. Before acid corrosion, both MFPB and NFPB can be well prepared to yield cubic shape with edge lengths of 200 and 166 nm, respectively. **b,** UV–Vis spectra of MFPB nanostructures as a function of the etching duration showing gradual appearance in NIR absorption following acid etching. **c,** UV–Vis spectra of NFPB nanostructures as a function of the etching duration showing gradual appearance in NIR absorption following acid etching. (One representative data was shown from three independently repeated experiments)

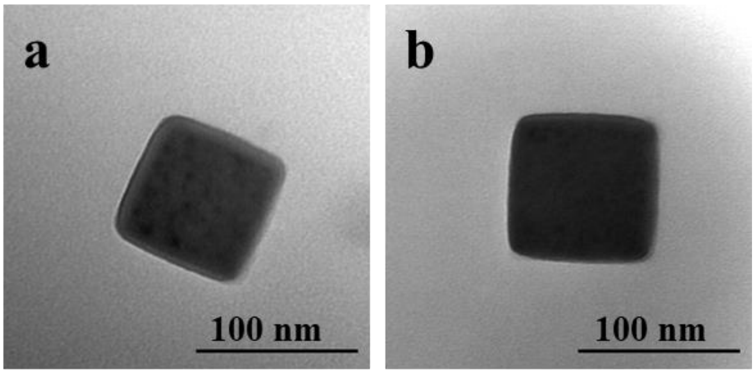

Supplementary Fig. 22 Stability test of the PB nanocrystal subjected to acid treatment using 0.01M HCl. a, b, TEM images of the nanocrystal before and after 24 h of acid etching, respectively. Resistance to corrosion was observed. (One representative data was shown from three independently repeated experiments)

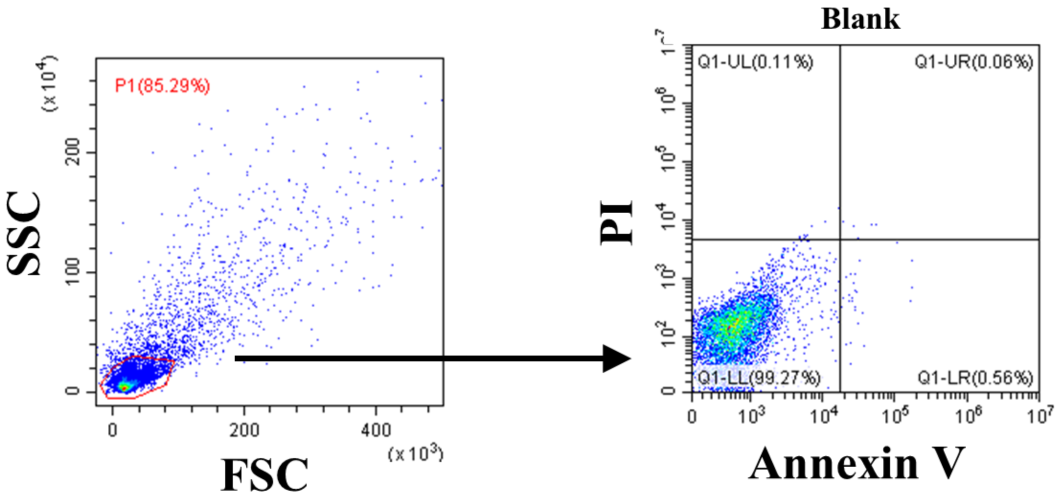

Supplementary Fig. 23 Figure exemplifying the gating strategy for HepG2 cancer cells for flow cytometry analysis in Fig. 5e.

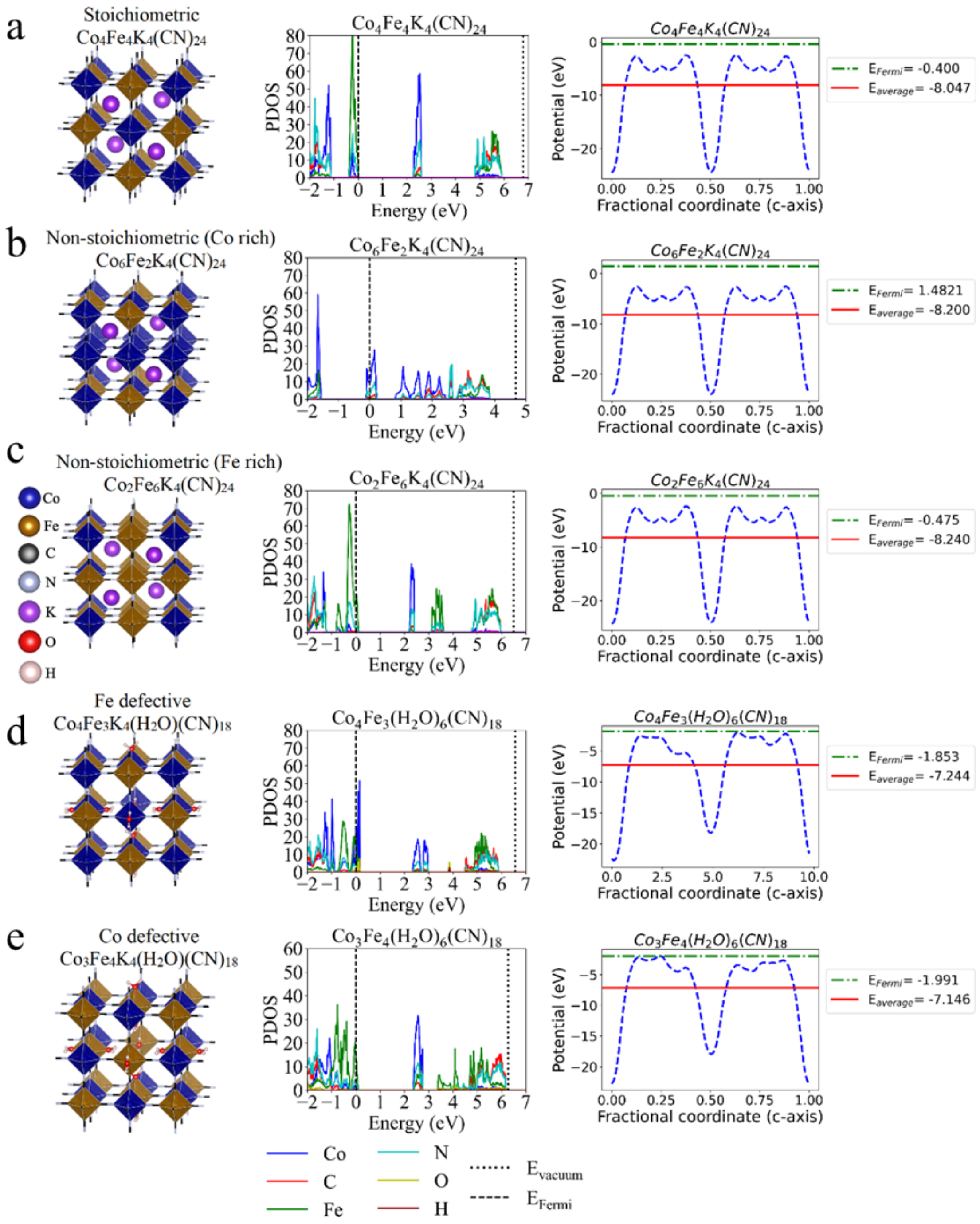

Supplementary Fig. 24 Atomic bulk structures of different CFPB and the corresponding projected density of states (PDOS) and electrostatic potential. a, Stoichiometric CFPB $Co_4Fe_4K_4(CN)_{24}$. **b,** Non-stoichiometric Co rich CFPB $Co_6Fe_2K_4(CN)_{24}$. **c,** Non-stoichiometric Fe rich CFPB $Co_6Fe_2K_4(CN)_{24}$. **d,** Fe defective CFPB $Co_4Fe_3(H_2O)_6(CN)_{18}$. **e,** Co defective CFPB $Co_4Fe_3(H_2O)_6(CN)_{18}$. The vacuum level ($E_{vacuum}$) in the PDOS plot is calculated from the surface structures (shown in Supplementary Fig. 21). Fermi level (E_{Fermi}) is calculated from Density Function Theory (DFT) calculations and shows the highest energy level that is occupied by electrons at zero K temperature. In the electrostatic potential plots (right), E_{Fermi} is at the same energy (potential) as PDOS and shown in the green dash-dotted line. $E_{average}$ is the average electrostatic potential across the bulk shown in the solid red line.

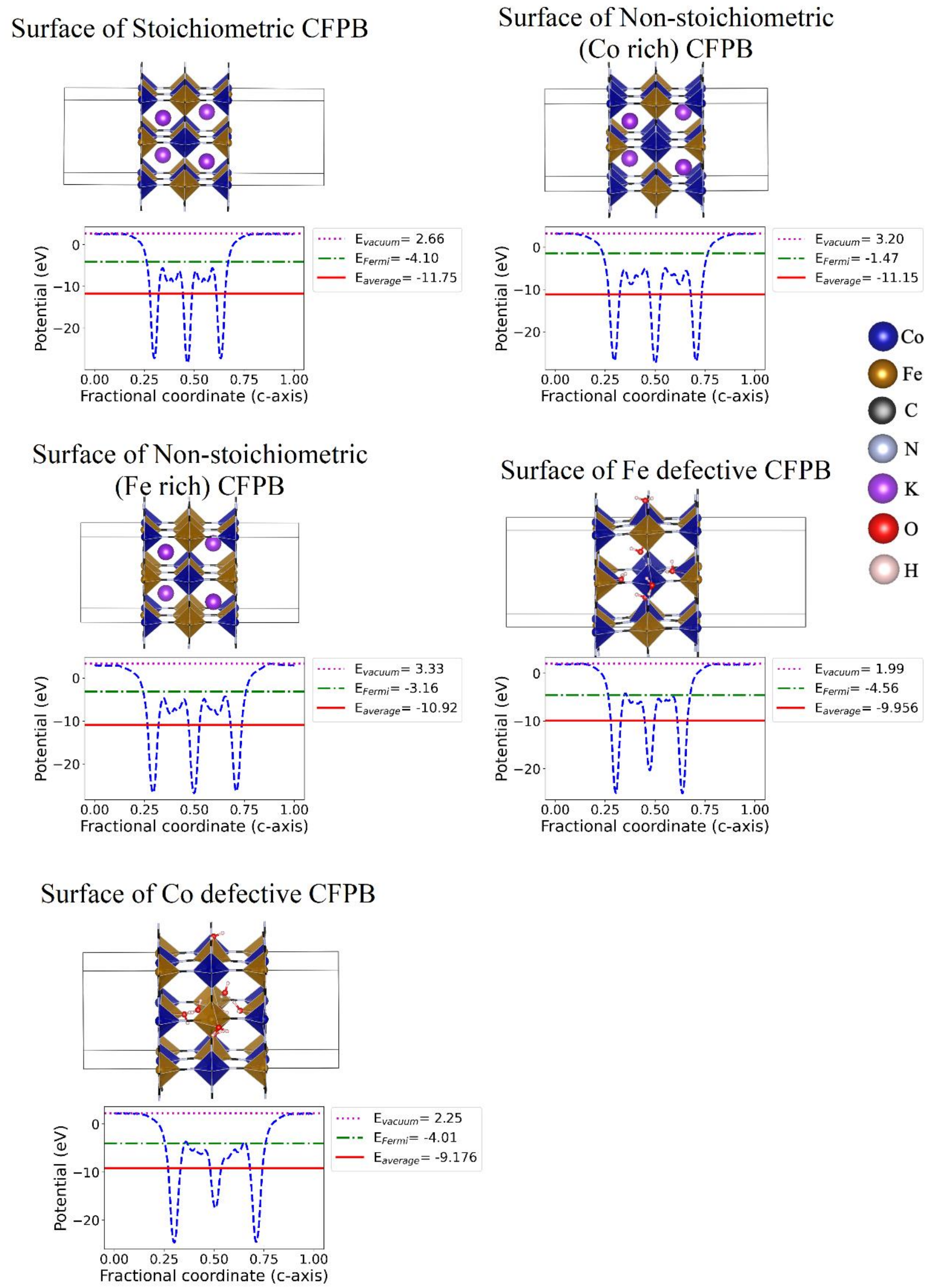

Supplementary Fig. 25 Atomic surface structures of different CFPB and the corresponding electrostatic potential from First-principles calculations. The solid red line is the average electrostatic potential ($E_{average}$) in the bulk-like region (slab), and the electrostatic potential in the vacuum (E_{vacuum}) is set at the highest potential in the vacuum region and labeled with the purple dotted line. The Fermi level in the surface structure is determined by adding the difference between the potential and Fermi level in bulk (shown in Supplementary Fig. 20**)** to the $E_{average}$. The work function can then be determined by the difference between the vacuum level and the Fermi level. This method was published in a previous study.[1]

Reference

(1) Ramprasad, R., Allmen, P. & Fonseca, L. R. C. Contributions to the work function: A density-functional study of adsorbates at graphene ribbon edges. *Physical Review B - Condensed Matter and Materials Physics* **60,** 6023–6027 (1999).
