# Supplementary material for: Prussian blue analog with separated active sites to catalyze water driven enhanced catalytic treatments
Source: Nat Commun. 2023 Aug 5;14:4709. doi: 10.1038/s41467-023-40470-z (PMC10404294; doi:10.1038/s41467-023-40470-z)
Supplement: Supplementary file 2 — Reporting Summary [file 41467_2023_40470_MOESM2_ESM.pdf]

nature portfolio

Corresponding author(s): Wei-Peng Li, Chia-Hao Su, Hong-Kang Tian, Chen-Sheng Yeh

Last updated by author(s): Jul 27, 2023

Reporting Summary

Nature Portfolio wishes to improve the reproducibility of the work that we publish. This form provides structure for consistency and transparency in reporting. For further information on Nature Portfolio policies, see our Editorial Policies and the Editorial Policy Checklist.

Statistics

For all statistical analyses, confirm that the following items are present in the figure legend, table legend, main text, or Methods section.

n/a	Confirmed	
☐	☒	The exact sample size (*n*) for each experimental group/condition, given as a discrete number and unit of measurement
☐	☒	A statement on whether measurements were taken from distinct samples or whether the same sample was measured repeatedly
☐	☒	The statistical test(s) used AND whether they are one- or two-sided<br>*Only common tests should be described solely by name; describe more complex techniques in the Methods section.*
☒	☐	A description of all covariates tested
☒	☐	A description of any assumptions or corrections, such as tests of normality and adjustment for multiple comparisons
☐	☒	A full description of the statistical parameters including central tendency (e.g. means) or other basic estimates (e.g. regression coefficient) AND variation (e.g. standard deviation) or associated estimates of uncertainty (e.g. confidence intervals)
☐	☒	For null hypothesis testing, the test statistic (e.g. *F*, *t*, *r*) with confidence intervals, effect sizes, degrees of freedom and *P* value noted<br>*Give P values as exact values whenever suitable.*
☒	☐	For Bayesian analysis, information on the choice of priors and Markov chain Monte Carlo settings
☒	☐	For hierarchical and complex designs, identification of the appropriate level for tests and full reporting of outcomes
☒	☐	Estimates of effect sizes (e.g. Cohen's *d*, Pearson's *r*), indicating how they were calculated

Our web collection on statistics for biologists contains articles on many of the points above.

Software and code

Policy information about availability of computer code

Data collection	The body weight of experimental mice were measured by electronic weighing machine (JENG HENG, Taiwan).<br>The blood biochemistry analysis for mice's serum were analyzed by FUJI DRI-CHEM 4000i (FUJIFILM, Japan).<br>The sections with H&E staining and IHC staining were observed and taken by microscope Olympus BX51 (Olympus, Japan).<br>The tumor growth of HepG2-Red-FLuc hepatocellular carcinoma cells in vivo were monitored by the Xenogen IVISR Spectrum Noninvasive Quantitative Molecular Imaging System (Caliper Life Sciences, USA).
Data analysis	The size of nanocubes was analyzed using the scan software. (version 5.0.0)<br>For flow cytometry analysis we used CytExpert. (version 2.5.0.77)<br>The bioluminescence of HepG2-Red-FLuc hepatocellular carcinoma within living body and isolated organs was analyzed by Living Image 4.7.3 (PerkinElmer, USA).<br>The statistical analysis of in vivo experiments was calculated by Origin 9.

For manuscripts utilizing custom algorithms or software that are central to the research but not yet described in published literature, software must be made available to editors and reviewers. We strongly encourage code deposition in a community repository (e.g. GitHub). See the Nature Portfolio guidelines for submitting code & software for further information.

Data

Policy information about availability of data

All manuscripts must include a data availability statement. This statement should provide the following information, where applicable:

- Accession codes, unique identifiers, or web links for publicly available datasets
- A description of any restrictions on data availability
- For clinical datasets or third party data, please ensure that the statement adheres to our policy

All data generated that support the findings of this study are present in the article and supplementary information. The full image dataset is available from the corresponding author upon request. Besides, source data are provided with this paper.

Human research participants

Policy information about studies involving human research participants and Sex and Gender in Research.

Reporting on sex and gender	n/a
Population characteristics	n/a
Recruitment	n/a
Ethics oversight	n/a

Note that full information on the approval of the study protocol must also be provided in the manuscript.

Field-specific reporting

Please select the one below that is the best fit for your research. If you are not sure, read the appropriate sections before making your selection.

[x] Life sciences [ ] Behavioural & social sciences [ ] Ecological, evolutionary & environmental sciences

For a reference copy of the document with all sections, see nature.com/documents/nr-reporting-summary-flat.pdf

Life sciences study design

All studies must disclose on these points even when the disclosure is negative.

Sample size	Sample size of in vivo experiments were chosen based on prior experience of the investigators with similar experiments previously published (ACS Applied Materials and Interfaces., 2022, 14(21), 24144-24159; Nat. Commun., 2022, 13, 7772; Advanced Material, 2019, 31(49), 1905087).
Data exclusions	No data was excluded from this study.
Replication	Results were replicated in independent experiments as described in manuscript.<br>Every experiments included replicates as describe in the figure legends and experimental methods.
Randomization	For in vitro study including cytotoxicity test, Live/Dead cells assay, apoptosis analysis and O2/H2O2/•OH detection, the same plates with equal seeding number of cells were randomized before treatment of different nanocubes. For in vivo study including biosafety test, biodistribution test and anti-tumor efficacy test, the experimental mice were randomized before implantation of carcinoma cells and administration of nanocubes or nanoframes.
Blinding	For in vitro study, the fields of Live/Dead cells assay and O2/H2O2/•OH detection were blindly taken. For other in vivo and in vitro study, the investigators needed to know the information of treatment group and the experimental results were presented objectively using respective instruments.

Reporting for specific materials, systems and methods

We require information from authors about some types of materials, experimental systems and methods used in many studies. Here, indicate whether each material, system or method listed is relevant to your study. If you are not sure if a list item applies to your research, read the appropriate section before selecting a response.

Materials & experimental systems

n/a	Involved in the study	
	x	Antibodies
	x	Eukaryotic cell lines
x		Palaeontology and archaeology
	x	Animals and other organisms
x		Clinical data
x		Dual use research of concern

Methods

n/a	Involved in the study	
x		ChIP-seq
	x	Flow cytometry
x		MRI-based neuroimaging

Antibodies

Antibodies used	IgG1 mouse monoclonal antibody was included in the Super Sensitive Polymer-HRP IHC Detection System (IgG1 is ready to use, #QD420-YIKE, BioGenex).<br>Antibodies used for IHC staining were phospho-histone H2A.X (Ser139) antibody (1:400 dilution, Invitrogen).
Validation	Antibodies were validated with different stained concentration and unstained group in tissue sections using IHC staining.

Eukaryotic cell lines

Policy information about cell lines and Sex and Gender in Research

Cell line source(s)	HepG2-Red-FLuc cells were obtained from PerkinElmer. A549 cells were obtained from BCRC. HUV-EC-C cell lines were obtained from ATCC.
Authentication	The cell lines used were not authenticated.
Mycoplasma contamination	The cell lines used were not tested for mycoplasma contamination.
Commonly misidentified lines (See ICLAC register)	No commonly misidentified cell lines were used in the study.

Animals and other research organisms

Policy information about studies involving animals; ARRIVE guidelines recommended for reporting animal research, and Sex and Gender in Research

Laboratory animals	Male BALB/c mice aged 4-7 weeks were obtained from the Laboratory Animal Center at National Cheng Kung University, Taiwan. NOD. CB17-Prkdcscid/NcrCrl (NOD-SCID) mice (6–8 weeks, male) were obtained from Labratory Animal Center, Kaohsiung Chang Gung Memorial Hospital, which is awarded Association for Assessment and Accreditation of Laboratory Animal Care International (AAALAC) accreditation. The experimental mice were housed in cages (three to five mice in each cage) at 22~23°C and 55 ± 10% humidity with 13 hr /11 hr light/dark cycle.
Wild animals	No wild animals were used in this study.
Reporting on sex	The sex difference was not important for in vivo experiments including biosafety study, biodistribution study and antitumor efficacy study. Therefore, in this study we performed the all animal experiments using male mice.
Field-collected samples	There are no samples collected from the field in this study.
Ethics oversight	Animal care was provided in accordance with the Laboratory Animal Welfare Act and the Guide for the Care and Use of Laboratory Animals and approved by the Institutional Animal Care and Use Committee of Kaohsiung Chang Gung Memorial Hospital (KCGMH). All animal treatments and surgical procedures were performed in accordance with the guidelines of KCGMH Laboratory Animal Center (IACUC NO. 2021031802).

Note that full information on the approval of the study protocol must also be provided in the manuscript.

Flow Cytometry

Plots

Confirm that:

☒ The axis labels state the marker and fluorochrome used (e.g. CD4-FITC).

☒ The axis scales are clearly visible. Include numbers along axes only for bottom left plot of group (a 'group' is an analysis of identical markers).

☒ All plots are contour plots with outliers or pseudocolor plots.

☒ A numerical value for number of cells or percentage (with statistics) is provided.

Methodology

Sample preparation	HepG2 cells were seeded in a 6-cm culture dish with a population of 5×10^5 cells and incubated overnight. Cells were then treated with CFPB nanoframes or CFPB@Lipo nanoframes at 28 ppm (in cobalt ion concentration). Control cells (culture medium only as negative control and 2 μM Thapsigargin as positive control) were also included in this experiment. After 24 h, cells were washed twice with PBS (phosphate buffer saline) and were later detached by trypsinisation. Then, the cells were harvested and washed with cold PBS. Cells were then re-suspended in 500 μL of 1X annexin V binding buffer. Next, 2 μL of annexin-V and 1 μL of PI (propidium iodide) were added to cells. Cells were gently vortexed, incubated at room temperature for 15 min and analyzed by flow cytometry (CytoFLEX S, Beckman Coulter).
Instrument	Beckman Coulter CytoFLEX S
Software	CytExpert
Cell population abundance	HepG2 cells were seeded in a 6-cm culture dish with a population of 5×10^5 cells and incubated overnight.
Gating strategy	Cell populations were initially gated using forward scatter and side scatter plot of cell only sample. The gate was set to remove dead cells and aggregates of cells and then applied to all samples.

☒ Tick this box to confirm that a figure exemplifying the gating strategy is provided in the Supplementary Information.
